# Influence of Polymer Composites and Memory Foam on Energy Absorption in Vehicle Application

**DOI:** 10.3390/polym12061222

**Published:** 2020-05-27

**Authors:** Ana Pilipović, Petar Ilinčić, Jelena Petruša, Zoran Domitran

**Affiliations:** Faculty of Mechanical Engineering and Naval Architecture, University of Zagreb, Ivana Lucica 5, 10000 Zagreb, Croatia; jelena_petrusa@hotmail.com (J.P.); zoran.domitran@fsb.hr (Z.D.)

**Keywords:** automotive industry, energy absorption, hybrid composites, memory foam

## Abstract

The automotive industry is one of the biggest consumers of polymer composites. Aside from good mechanical properties, polymer composites have low mass, which positively affects the overall vehicle weight reduction and improves energy efficiency. Although polymer composites are used in various vehicle components, this paper focused on the application in vehicle bumper production. Two different composite plates with hybrid fiber layup were made; the first plate with a combination of glass and carbon fibers and the second with carbon and aramid. For comparison, and as a cheaper variant, a third plate was made only with glass fibers. In the first two plates, epoxy resin was used as the matrix, while in the third plate, polyester resin was used. Polyurethane memory foams of different densities (60, 80, 100 kg/m^3^) and thicknesses (10, 15, 20 mm) were used as impact force energy absorbers. With the factorial design of experiments, it was found that the thickness of the memory foam was the main influence factor. Without the use of memory foam, the hybrid composite, made of glass and carbon fibers, showed the highest energy absorption, while with the use of foam, the highest energy absorption was achieved with the glass fiber composite. Without the memory foam, the impact force measured on the glass/carbon hybrid composite was 9319.11 ± 93.18 N. Minimum impact force to the amount of 5143.19 ± 237.65 N was measured when the glass fiber composite plate was combined with the memory foam. When using memory foam, the impact force was reduced by 30–48%, depending on the type of composite used.

## 1. Introduction

Passenger safety, exhaust emissions, and fuel efficiency are the most important issues in modern vehicle design. One of the ways to increase fuel efficiency and reduce exhaust emissions is the reduction of overall vehicle mass. Fiber-reinforced polymer composite materials can contribute to this reduction in mass, while meeting the automotive industry demands such as mechanical properties, durability, corrosion resistance, lightweight, energy absorption, vibration and noise reduction [1,2,3].

According to [4,5], about a 25% reduction in car mass would be equivalent to saving 250 million barrels of crude oil, while every 10% of reduction in weight increases the fuel economy by 6–8%. Weight reduction can be achieved on different vehicle parts, especially the structural parts. The automotive bumper beam as a structural component absorbs up to 80% of kinetic energy during a collision, thus protecting the passengers and the car body. It is located on the front and rear ends of a vehicle, usually hidden behind plastic or metal fascia [6,7]. About 70% of all impacts occur in the front of the car (35% front, 15–20% front left and right side) and 14% at the rear end (10% rear, 2% rear left and right side) [8].

**Figure 1:**
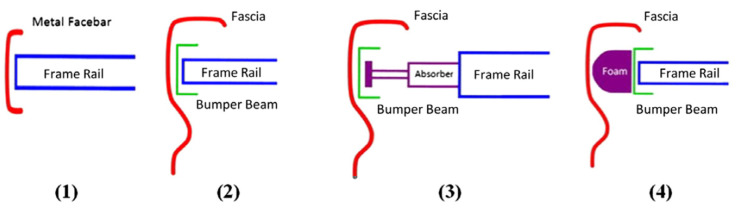
Common bumper system reproduced from [9] with permission from Elsevier, 2012.

There are four bumper systems in common use today, as shown in Figure 1 [9], but the main components of an automotive bumper beam are a horizontal reinforcing beam and energy absorber in the form of a crush box, foam, honeycomb, axial beam, or axial mechanical absorber.

With the optimized design and appropriate material selection of the metal elements that connect the bumper with the chassis, the specific energy absorption can be enhanced from 194% for steel to 225% for magnesium alloy with aluminum foam compared to the conventional element design [8]. Schmidova et al. conducted a dynamic impact test using drop weight on the composite absorbers (carbon fiber-reinforced epoxy resin in the form of prepregs) in the shape of tubes. Carbon fiber was in the 0° layer orientation. Maximal observed deformation energy was 2885 J [10]. A simulation of carbon fiber composite bumper with the finite element method was carried out by the authors Godara, S.S. and Nagar, S.N. They simulated a vehicle frontal bumper beam with eight different cross-sections and concluded that even different forms of bumper changed the stress in the range from 990 to 2700 MPa for the impact speed of 30 km/h [11].

Energy absorbers in the form of foam, honeycomb, or mechanical absorber are very useful in the case of low-speed impact because after the impact, the bumper returns to its original position. Foam and honeycomb absorbers are usually made from polymers like polypropylene, low-density polyethylene, or polyurethane. Mechanical energy absorbers are mostly made of metal in the form of crush cans, axial beam, or shock absorber. Their mass is several times higher than the mass of polymer energy absorber, but their energy absorption capacity is also several times higher. At a low-speed impact, a mechanical absorber should deform in an elastic region and together with the elastic bending of the bumper beam absorb the energy. After the impact, the elastic deformation enables the bumper to return to its original position. In the case of high-impact collisions, the mechanical absorber and bumper beam absorb the impact energy by plastic deformations. After plastic deformation, the excess of impact energy is transferred and absorbed by the deformations of the front frame (frame rail) and the body main-frame [3,4,9,12,13].

Due to high energy absorption, most of the front bumper systems use some form of mechanical energy absorbers, but to increase the vehicle safety and energy absorbing capabilities of the bumper systems, new materials, shape geometries, and production technologies are required.

With that in mind, authors Mo, F. et al. developed a new geometry of the front bumper energy absorber by changing the deformation level of the X-shaped energy absorber. During the impact, the absorbed energy level is variable and depends on the amount of external energy applied. This kind of design allows high pedestrian safety and good low-speed impact capability [14].

Aside from geometry, new materials are also being developed for bumper systems. Along with energy absorption, new materials have also focused on lightweight design. For this purpose, polymer composites have been investigated as an alternative bumper system material. The importance of using polymer composites in the automotive industry is their high specific modulus and strength, good thermal stability, durability, stiffness, flexural properties, impact strength, corrosion resistance, and good formability [15,16,17]. In studying the composite materials, special attention has been paid to hybrid composites [18]. In fiber hybridization, two or more types of fiber are combined in a matrix to mitigate the disadvantages of the type of fiber, keeping the benefits of others. The synergistic effects of mixing the fiber aids in enhancing the properties of the composite material that neither of the constituents have [17]. Hybrid composites are composites in which the material properties are tailored according to specific requirements. With the flexibility of the material properties’ shaping, it is possible to achieve a balance between high performance and optimal costs of hybrid composites. Therefore, they are applicable in high-load structural applications like structural parts in the automotive industry [19].

For a lightweight bumper beam design, different types of composite materials like carbon fiber-reinforced plastic (CFRP), glass fiber-reinforced plastic (GFRP), sheet molding compound (SMC), and glass mat thermoplastic (GMT) have been investigated and tested. Better mechanical properties are achieved with CFRP and GRRP materials, but SMC and GMT materials are widely used because of their lower material and production costs [20,21,22,23]. For such reasons, the material development for bumper systems should be directed toward hybrid composites.

Tailor-made performance materials can also be seen in the study made by Neale et al., where 3D woven composites were used for automotive crashworthy structures. From the test results, it was concluded that with the selection of an appropriate pick density of 3D woven carbon fibers, specific energy absorption could be increased in the static test from 84 J/g up to 104 J/g and in the dynamic test from 70 J/g up to 93 J/g [24].

According to Wang et al., there are numerous advantages of composite materials compared to steels for automotive application. Above all, the use of composites can result in the weight reduction of 20–40%. Other advantages are the styling flexibility, 40–60% reduced tooling cost, reduction in assembly costs and time, resistance to corrosion and scratches, reduced noise vibration harshness (NVH), and higher damping, and higher specific energy absorption [5].

In their study, Díaz-Álvarez et al. used bio-composites for the production of bumper beam (PLA/flax) and reported that the absorbed energy increased with the impact energy increase. In the case of impact energies of 30 J and higher, the peak value of force was about 3500 N, but failure occurred (the fiber cracked) [25].

As above-mentioned, in the automotive industry, foam, and honeycomb absorbers are made of different materials like polypropylene, polyurethane (PUR), or low-density polyethylene. Various types of PURs can be found on the market since it is formed of two components, isocyanates and polyols. Depending on the type of monomer and other additives for properties shaping, PUR is produced in different densities and hardness. In modern vehicles, an average of 20 kg of all types of PUR can be found in a single car [26,27]. PUR can be used for the exterior and interior, especially for the elimination of vehicle noise [28] and with the use of additives, flame resistance can be improved [29]. Linul et al. investigated the influence of aluminum micro-fibers (AMs) on the compression properties and energy absorption of PUR. They found that by adding 1.5% AMs to PUR, compression strength increased 61.81% and energy absorption increased 71.29%, but above 1.5% of AMs energy absorption was only 12.68% and compression strength decreased. The energy absorption was also dependent on the percentage of micro-fiber in the same manner as the strength properties [30].

In this research, memory foam was used, which is a type of PUR foam, but usually has a higher density and is more expensive than the regular PUR foam [31].

Flexible polyurethane foam is a visco-elastic material that has special mechanical properties like high energy absorption, low density, and low stiffness. The automotive industry uses soft polyurethane foam as a major element in the production of modern automotive seats. Aside from non-linearity and visco-elasticity, soft foam also has a memory effect of past loading history and requires time to recover to its original form [32].

Shape-memory materials have the ability to store the deformed shape and recover to its original shape (pre-deformed). This phenomenon is usually caused by temperature change and has been observed in metals, ceramics, and polymers. Shape-memory polymers are polymers with a chemically or physically cross-linked network structure like a thermoset or thermoplastic [33,34,35].

Standard foams have a pressure strength from 3 to 4 kPa, meaning that they provide a return force (spring effect) when they are exposed to pressure. Due to their memory shape, memory foams, unlike standard foams, have up to 4x lower compressive strength and do not produce as much return pressure [36].

The properties of memory foam are density 80 kg/m^3^ (+/− 10%), tensile strength 0.15 N/mm^2^, deformation up to 5%, elasticity 10%, and recovery time after deformation of about 10 s [36].

Memory foam is produced by block casting or by direct molding into the desired shape. Block casting is more economical but can be used only for the production of foams with a density from 50 to 60 kg/m^3^. The main disadvantage of low-density foams is that persistent foam deformation occurs with aging, which is manifested in the loss of thickness and the sudden loss of the “memory” effect. With direct molding, densities up to 100 kg/m^3^ can be produced, resulting in greater resistance to permanent deformations and the memory effect lasts longer than the one of the block casting. For high quality memory foam, molding is the preferred process [37].

By reviewing the literature, it can be concluded that the majority of authors in their works have dealt either with different bumper designs (metal or composite) or with the elements that connect the bumper to the vehicle chassis. With various forms of such coupling elements, attempts were made to obtain the reductions of the impact energy. In some of the tests, classic energy-absorbent materials such as ordinary PUR, polyethylene (PE), polypropylene (PP), polystyrene (PS) were used.

Unlike previous studies, the aim of our study was to test the energy absorption properties of different polymer fiber-reinforced composite plates combined with memory foam intended for the vehicle bumper system. The backbone of the work is the application of hybrid composites, because the use of hybrid composites will not only reduce the cost of the currently most used single-component carbon fiber composite, but also identify how the advantages of different materials combined together can contribute to the overall material property improvement.

In this research, two different composite plates with a hybrid fiber layup were made: the first plate with a combination of glass and carbon fibers, and the second one with carbon and aramid fibers.

In order to compare the energy absorption properties, a glass fiber plate was also made. Due to the lower price of glass fiber and polyester resin, this plate was investigated for potential use as a cheaper alternative composite in the automotive industry application.

Polyurethane memory foam of different thicknesses and densities was used in combination with the composite plates to test the possibilities of the (additional) absorption of impact energy.

Impact force analysis of different composite materials, thickness, and density of memory foam was performed with a factorial design of experiments.

Three test plates were made for each type of composite (glass fiber, glass + carbon fibers, and carbon + aramid fibers), or a total of nine plates. In this way, the tests were repeated to obtain the results for the mean value and the standard deviation.

## 2. Materials and Production of Hybrid Composites

**Figure 2:**
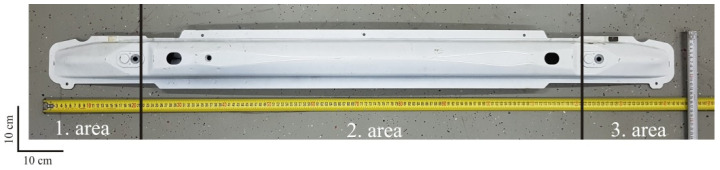
Steel car bumper with marked areas of measurement.

The thickness of the test plates for the composite bumper was determined according to Prabhakaran, S. et al. [1] from the properties of the metal bumper taken from the production passenger car (Figure 2).

Therefore, the moment for steel is:

(1) $$\frac{M}{I}=\frac{R_{m}}{y}$$

where *M* (Nm) is the bending moment; *I* (m^4^) is the moment of inertia; *R*_m_ (N/m^2^) is the tensile strength; *y* = *d*/2, *d* (m) is the thickness of the bumper.

Moment of inertia for the rectangular section is:

(2) $$I=\frac{b\cdot d^{3}}{12}$$

where *b* (m) is the breadth of the bumper. There were three sections in the bumper, *I*_1_, *I*_2_, and *I*_3_, respectively.

Dimensions of the bumper thickness and width were measured on the steel bumper of the car (Figure 2): *d* = 0.002 m, *b*_1_ = 0.025 m, *b*_2_ = 0.091 m, and *b*_3_ = 0.025 m.

The bumper was divided into three areas, so accordingly, there were three moments of inertia:

(3) $$I_{1}=I_{3}=\frac{0.025\times 0.002^{3}}{12}=1.7\times 10^{-11}m^{4}$$

(4) $$I_{2}=\frac{0.091\times 0.002^{3}}{12}=6.1\times 10^{-11}m^{4}$$

(5) $$I=I_{1}+I_{2}+I_{3}=7.8\times 10^{-11}m^{4}$$

Tensile strength of steel = 460 × 10^6^ N/m^2^ [1]. The *y* value was 0.002/2. By inserting the value into Equation (1), the value of the moment was *M* = 35.88 Nm.

The test plate thickness was determined based on the calculated moment and the tensile strength for each type of composite by inserting the inertia moment and the tensile strength values for the glass fiber composite *R*_m_ = 360 × 10^6^ N/m^2^ [38] in Equation (1):

(6) $$\frac{35.88}{\frac{0.025\times d_{1}^{3}}{12}}=\frac{\;360\times 10^{6}}{\frac{d_{1}}{2}}$$

*d*_1_ = *d*_3_ = 4.88 × 10^−3^ m,

(7) $$\frac{35.88}{\frac{0.091\times d_{2}^{3}}{12}}=\frac{\;360\times 10^{6}}{\frac{d_{2}}{2}}$$

*d*_2_ = 2.56 × 10^−3^ m,

(8) $$d=\frac{d_{1}+d_{2}+d_{3}}{3}=4.11\times 10^{-3}m$$

Repeating the calculation process (Equations (1)–(8)) for the composite plate of carbon and aramid fibers with a tensile strength of 404.3 × 10^6^ N/m^2^ [39], and the calculated thickness *d* was 3.89 mm.

For the composite made from carbon and glass fiber with a tensile strength 440 × 10^6^ N/m^2^ [40], the calculated thickness *d* was 3.7 mm.

Based on the calculated values, it was taken that the thickness of the composites test plates would be *d* = 4 mm.

### 2.1. Materials for Composite Plates

The first test plate was made of glass fiber and polyester resin. The glass fibers were woven into a triaxial fabric 0°/45°/45°, meaning that the fibers were oriented in three directions. The thickness of the fabric was 0.6 mm. This combination was chosen since glass fibers are the least expensive and polyester resin is the most commonly used commercial glass fiber resin.

The second test plate was made of carbon and aramid fibers and epoxy resin according to our previous research for application in the transport industry [39]. The carbon fiber fabric was plain woven (thickness: 0.2 mm; fabric surface mass: 200 g/m^2^) and the aramid fiber fabric was satin woven (thickness: 0.18 mm; fabric surface mass: 170 g/m^2^). Since the best mechanical properties of the composites are achieved when the forces and stresses act in the direction of the fibers, the outer carbon fibers were oriented in the longitudinal direction 0° and 45° direction, while the inner aramid fibers were oriented only in the longitudinal direction 0°. Aramid fibers were placed as an inner layer of the composite to increase the impact strength [39].

The third test plate was made of carbon and glass fibers and epoxy resin according to the research and the results given in a paper by Song [40]. The plain woven carbon fiber fabric and triaxial glass fiber fabric were arranged in the composite in four layers: the first and the third layer were carbon fibers, and the second and the fourth layer were glass fibers. This way of layup resulted in the best flexural properties of the composite, but the acting force had to be applied on the glass fiber side [40]. Therefore, in this paper, the glass side was oriented as the outer side of the bumper.

**Figure 3:**
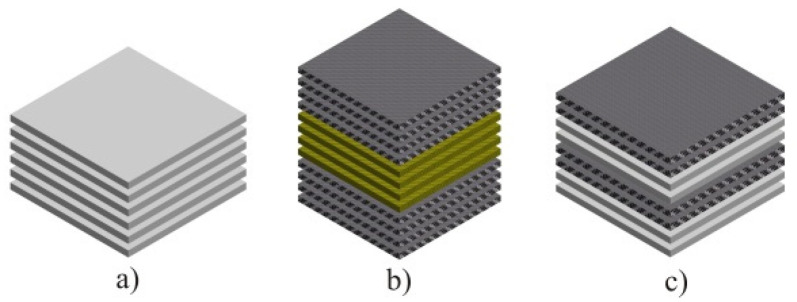
Laminating structure: (**a**) glass plate, (**b**) carbon/aramid plate, (**c**) glass/carbon plate.

Figure 3 shows the arrangement of the layers in the composite plates, according to Table 1.

To achieve the calculated thickness of 4 mm, composite plates were made with a different number of fabric layers, according to Table 1.

Reinchhold polyester resin NORPOL 440–800 crosslinked with 2% PROMOX P200TX was used as the matrix for glass fibers. Two other hybrid composite plates were made with epoxy resin HEXION™ Specialty Chemicals L285 in combination with the crosslinking agent HEXION™ Specialty Chemicals H286 in mass fraction 2.5 to 1. The gel time was about 40 min, while the complete curing time took 24 h [41,42].

### 2.2. Production of Composites Plates

**Figure 4:**
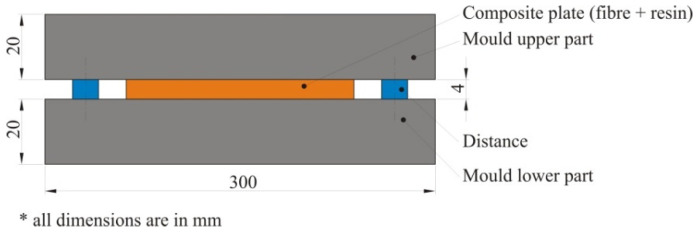
Mold for the production of composite plates.

The test plates were made with a combination of the hand lay-up and compression molding process. For the production of one glass fiber test plate with the dimensions 200 mm × 200 mm, 150 g of resin and 3 g of crosslinking agent were required, while for the same dimension of a hybrid test plate, 150 g of resin and 60 g of crosslinking agent was required. The mold was made of two metal plates (upper and lower plate) divided by 4 mm thick distances between the plates as shown in Figure 4. The upper and lower mold parts were tightened with screws to create the required pressure.

**Figure 5:**
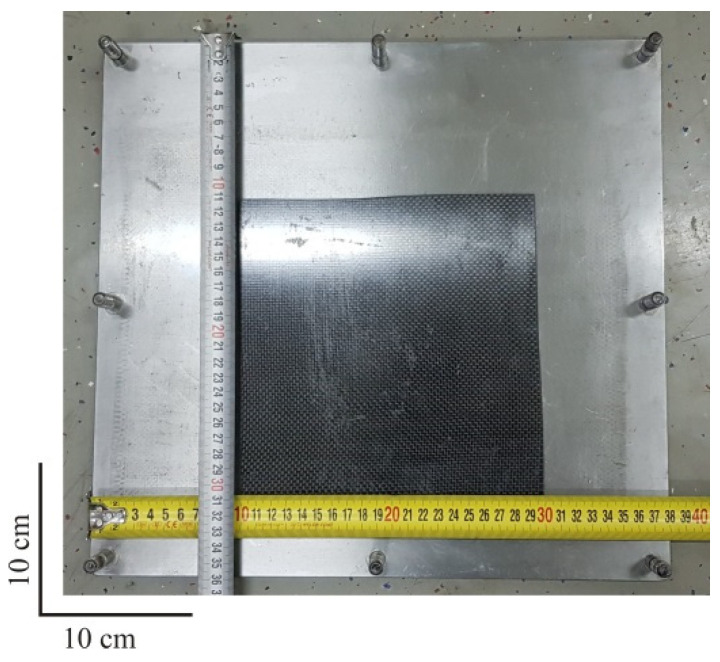
Finished composite plate on the lower half of the mold.

First, a release agent was applied to both inner parts of the molds, which at the end of the production process enabled an easier separation of the finished composite plates from the mold. On such a prepared mold, fiber fabrics were laid up according to Table 1 (Figure 5). After the lamination process was completed, the mold was closed and placed at a temperature of 30 °C for 24 h to cure the resin. After demolding, the plates were cut to dimensions of 200 mm × 200 mm.

According to the factorial design plan, the foams with a density of 60 kg/m^3^, 80 kg/m^3^, and 100 kg/m^3^ in three different thicknesses of 10 mm, 15 mm, and 20 mm were used for the absorption of impact energy.

## 3. Description of an Absorption Test

**Figure 6:**
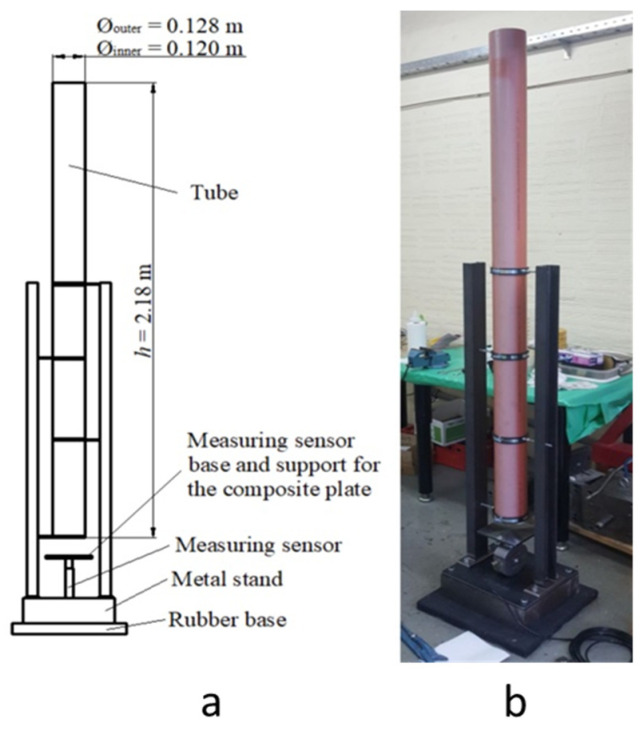
Test setup: (**a**) scheme, (**b**) real photo.

Based on other research and studies [23,43,44,45], the energy absorption of composite plates and memory foam was tested in a test similar to the drop dart test. The test setup was our own and it consisted of a rubber base, a metal frame with polymer tube, and a measuring pad with a force transducer. The test setup is shown as a scheme in Figure 6a and a photo in Figure 6b.

The test plate and the memory foam were placed on a measuring pad, whose center coincided with the centerline of the polymer tube. A flat bottom weight with a mass of 2.64 kg and a diameter of 117 mm was dropped from a defined height through the polymer tube on the composite plate and the memory foam whereby the impact force was measured. The force transducer with the measuring range 100–100,000 N and an accuracy <0.03 was connected to the *HBM Spider* data acquisition system.

Weight drop height was adjusted to the 2.085 m for the impact energy to be equal to 54 J:

(9) E=m·g·h=2.64×9.81×2.085=54 J

where *E* is the energy (J); *m* is the mass (kg); *g* is the gravity acceleration (m/s^2^); and *h* is the height (m).

**Figure 7:**
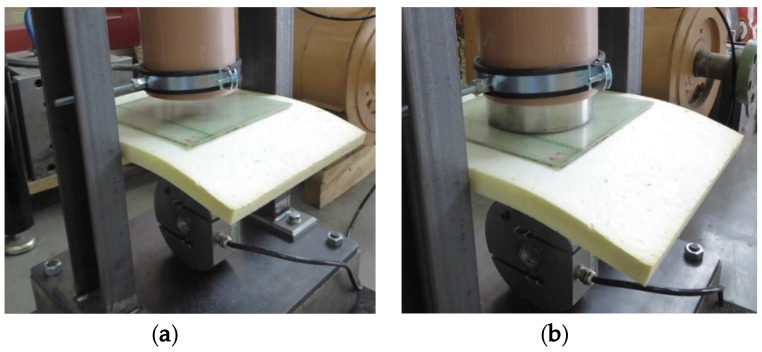
Testing: (**a**) before the test, (**b**) after the test—after the weight is dropped.

In all of the tests (measurements), the weight hit the composite plate first (Figure 7). The decision to hit the composite plate first instead of the memory foam was determined by the pre-tests where lower impact forces were measured in this configuration and by the fact that on the car bumper, the composite part would be external and the absorber would be placed on the inner side of the bumper [9].

## 4. Results of Absorption Test

Two level factorial design with three center points per block was used for tests to obtain the repeatability of the results. Each type of the composite test plate was tested with seven different memory foams, depending on the density and the thickness according to the factorial design of the experiment. For each combination, the measurement was repeated five times to obtain the mean value of the test results. Additionally, to compare the initial impact force and the energy absorption of the composite test plates only, each type of the composite plate was also tested without the use of the memory foam. The test results for all three types of composite plates and memory foam combinations are shown in Table 2, Table 6 and Table 10.

The test found that the impact force was influenced by two parameters: density (Factor A) and the thickness of the memory foam (Factor B). Software package *Design Expert* and the module ANOVA (analysis of variance) were used to analyze the test results.

### 4.1. Results for Glass Fiber/Polyester Composite

The results of the measured impact force with the mean values and standard deviation for glass fiber/polyester resin and memory foam are given in Table 2. The results of run 3 were excluded from the analysis due to the excessive deviation from the other center points (condition of experiments in runs 1 and 5).

The results of the variance analysis for the first test plate (glass fiber/polyester resin) and memory foam are shown in Table 3.

For a certain factor to affect the change, the Prob > *F* value should be smaller than 0.05 [46]. It can be concluded from the table that factor A, the density of the memory foam, did not have a significant influence on the change of the impact force, while factor B, the thickness of the memory foam, had a significant influence on the force change. Likewise, the deviation from the model (curvature) was insignificant, which meant that the selected model was appropriate.

The basic statistical data about the model are given in Table 4. R-squared (*r*^2^) is the measure of deviation from the arithmetic mean, which is explained by the model. The closer *r*^2^ is to 1, the model better follows the data. The calculation of R-squared is made according to Equation (10): [46]

(10) $$r^{2}=1-\frac{{SSD}_{\mathrm{residual}}}{{SSD}_{\mathrm{model}}+{SSD}_{\mathrm{residual}}}$$

where *r*^2^ is the R-squared and *SSD* is the sum of square deflection.

From Table 4, it can be concluded that the model followed the data very well since the coefficient of determination was *r*^2^ = 0.9992.

The standard error and confidence intervals (CI) are given in Table 5. The standard error represents the standard deviation of an estimate coefficient (i.e., it is a measure of statistical accuracy), which must be a positive value. The design matrix in this experiment was orthogonal, so that the standard error for each estimated coefficient was the same. The confidence interval uses the estimated standard errors to draw an interval with a certain level of confidence around the estimated effect. A 95% confidence interval means that the true estimate of the population parameter will lie in the interval 95 out of 100 times. For more information, see the literature on the design of the experiment.

**Figure 8:**
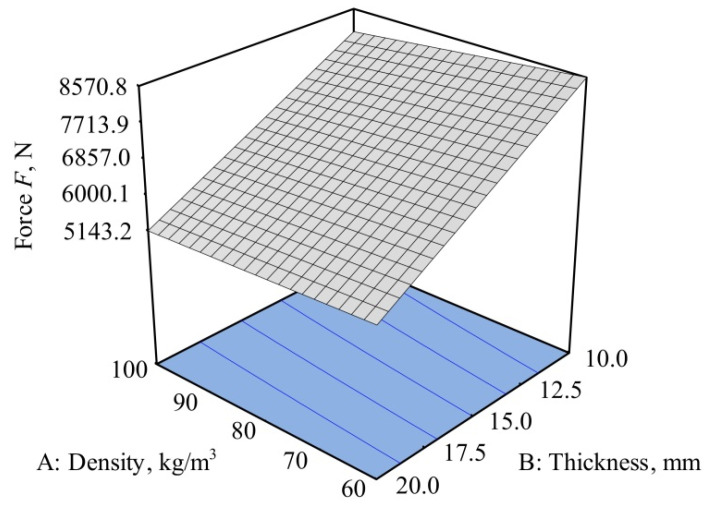
Dependence of the density and thickness of the memory foam on the impact force for the glass fiber/polyester composite.

The dependency of the density and thickness of the memory foam on the impact force for the glass fiber plate is shown in Figure 8. The highest reduction of impact force was observed at a foam density of 100 kg/m^3^ and a thickness of 20 mm.

Figure 8 also shows that the influence of factor A, the density of the memory foam on the force reduction, was relatively small compared to factor B, the thickness of the memory foam, which was dominant.

From the analysis model for the impact force, *F* can be described by the equation:

(11) F=13203.85−23.58·density−376.84·thickness+0.92·density·thickness

### 4.2. Results for Carbon/Aramid Fiber/Epoxy Hybrid Composite

Table 6 presents the results of the measured impact force with the mean and standard deviation for carbon and aramid fiber/epoxy resin and memory foam. One single test result for memory foam 80/15 was rejected in runs 3 and 5 because they deviated from the other results and were not considered when calculating the mean value.

Table 7 gives the analysis results for the second test plate (carbon and aramid fibers with epoxy resin) and memory foam, and Table 8 shows the basic statistical data for the model of impact force on the second test plate.

From the given data in Table 8, it can be concluded that the model followed the data very well. The coefficient of determination is *r*^2^ = 0.9839, which is slightly lower than the determination coefficient for the first glass fiber test plate. The values of standard errors are shown in Table 9.

**Figure 9:**
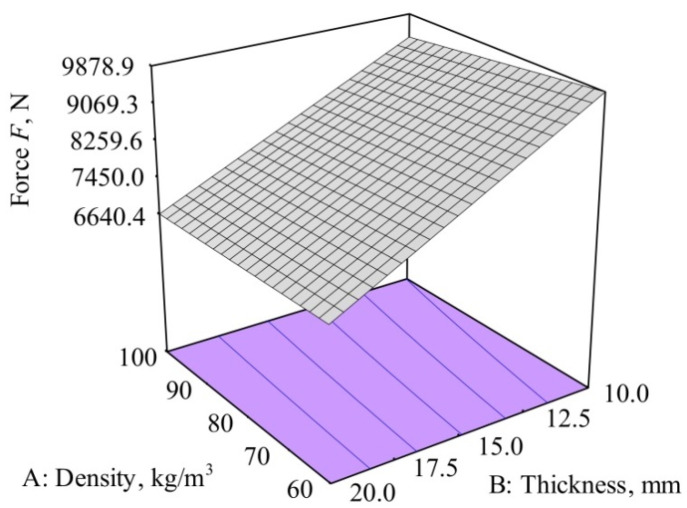
Dependence of the density and thickness of the memory foam on the impact force for the carbon/aramid/epoxy hybrid composite.

Figure 9 shows the force dependence on the density and thickness of the memory foam for the second test plate (carbon and aramid fibers with epoxy resin). The highest reduction of impact force was observed, like in the case of the first plate, at a foam density of 100 kg/m^3^ and foam thickness of 20 mm. Factor B, the thickness of the memory foam, had a significant effect on the force change, while the influence of factor A, the density of the memory foam, on the force change was very small. It was also shown that factors AB did not affect the change in the impact force.

Just like for the first plate, the deviation from the model for the second plate was also not significant.

The model for the impact force *F* can be described by Equation (12):

(12) F=14460.46−24.05·density−378.52·thickness+1.08·density·thickness

### 4.3. Results for Carbon/Glass Fiber/Epoxy Hybrid Composite

The results of the measured impact force with the mean and standard deviation for the carbon and glass fiber/epoxy resin and memory foam are given in Table 10.

The results of the analysis for the third test plate (hybrid carbon/glass fiber with epoxy resin) and memory foam are shown in Table 11.

Table 12 gives the basic statistical data for the model of impact force on the third test plate (carbon/glass/epoxy hybrid composite) and memory foam. With the coefficient of determination *r*^2^ of 0.9947, the model fit the data very well.

The values of the standard errors are shown in Table 13.

**Figure 10:**
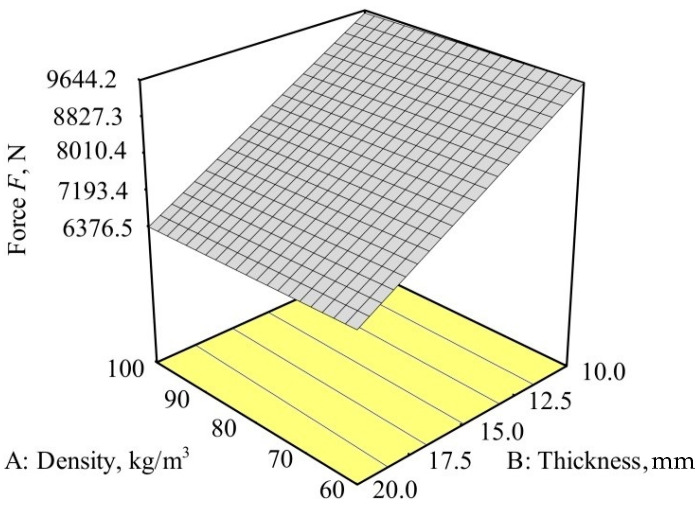
Dependence of the density and thickness of the memory foam on the impact force for the carbon/glass/epoxy hybrid composite.

The influence of the density and thickness of the memory foam on the impact force for the third test plate (carbon/glass/epoxy) is shown in Figure 10. The highest reduction of impact force was again observed at a foam density of 100 kg/m^3^ and thickness of 20 mm. Like in the previous cases, the dominant factor on the impact force change was factor B.

For the third test plate, the model for the impact force *F* can be described by the equation:

(13) F=12777.62−0.10·density−304.04୽thicknes−0.17·density·thickness

### 4.4. Discussion of the Results

After energy absorption, analyses were carried out to determine which composite was the most appropriate for use in the automotive industry, and the weight of the composite was also considered. Masses and thicknesses of each type of composite test plate were measured after the final cutting to the required dimension of 200 × 200 mm. Mean mass of the glass/polyester plate was 351.96 ± 4.62 g and the mean thickness was 4.30 ± 0.09 mm. For the carbon/aramid/epoxy plate, the mean mass was 235.87 ± 2.31 g and the thickness was 4.13 ± 0.05 mm. The mean mass of the carbon/glass/epoxy plate was 295.63 ± 0.39 g and the thickness was 4.06 ± 0.04 mm.

Thicknesses of all test plates were in the range from $4_{+0.3}^{+0.06}$ mm, which satisfied the required minimal thickness of 4 mm calculated from the metal bumper dimensions (Equations (1)–(8)).

**Figure 11:**
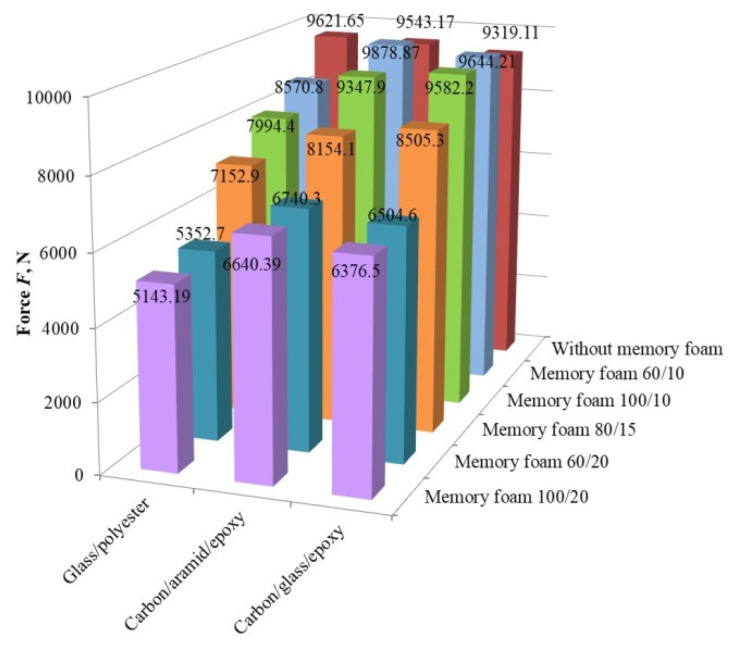
Comparison of the force results without and with memory foam.

The comparison of the results of the impact force for all three types of test composite plates without and with the use of memory foam is shown in Figure 11. Without the use of memory foam for the first (glass fiber and polyester resin) test plate, the highest impact force of *F* = 9621.65 ± 404.58 N was measured, meaning that the glass fiber test plate had the lowest energy absorption capacity. The third (carbon and glass fiber) test plate showed the best absorption properties with the lowest impact force (*F* = 9319.11 ± 93.18 N) measured. Such a result was expected since the carbon fibers had the highest specific absorption energy, which was shown in studies by Lukaszewicz, D. and Fazita, N.M.R. et al. [47,48].

The difference between highest (glass fiber) and lowest (carbon/glass) measured impact forces was only 3.2%, which is in line with that given in [40]. Song, J. tested different types of hybrid glass/carbon and aramid/carbon composites and concluded that the fracture energies of these composites were similar. Likewise, the results of the modulus of elasticity and tensile strength of different aramid/carbon hybrids were within 3–4%.

Results of the impact force measured for the glass fiber composite without the memory foam can be compared with similar studies like that by Ramyasree et al. [6]. It can be observed that for the glass mat thermoplastic composite used in their research, the impact force was 15 kN, which was 56% more than the force measured for the glass composite plate in our study. For comparison, Ramyasree et al. stated that for the steel bumper impact force was 60 kN. However, it should be mentioned that in the study by Ramyasree et al. (2015), a simulation was used without any real tests. These are in contrast to the results given by Díaz-Álvarez et al. [25], who studied the use of bio-composites for the production of bumper beam (PLA/flax). In their research, several tests with different impact energy levels were made and it was concluded that the absorbed energy increased with the increase of the impact energy. In the case of impact energies of 30 J and higher, the peak value of force was about 3500 N, but failure occurred (the fiber cracked) [25]. Such results are almost three times smaller than the results obtained in our research, but it should be noted that they used bio-composites, which are known to be a material with a lower mechanical property. Additionally, the shape of the impactor is quite different. In the study by Díaz-Álvarez et al. [25], the impactor had a Charpy shape nose with a diameter of 20 mm, while in our research, the impactor was in the shape of a flat cylinder with a diameter of 117 mm. For better comparison of the results, the tests should be made with the same testing equipment.

If the test plates are compared by production costs, the third composite plate was more expensive than the first glass plate due to the use of carbon fibers, while the second plate was the most expensive because of the use of carbon and aramid fibers.

When comparing the results of the impact force for all three types of test composite plates with the use of memory foam, it can be concluded that only the thickness of the memory foam had a significant influence on the change in the force. Such results are in line with the results given in Sounik et al. [49], who investigated the properties of polyurethane energy-absorbing foams and concluded that for the foam density of 80 kg/m^3^ and three different thicknesses (13, 25, 64 mm), the best energy absorption was achieved with the thickness of 64 mm. Since the energy absorption comes from the compression of the foam, energy is absorbed through a combination of elastic deformation and pneumatic effects [49]. Energy in polymeric foams is dissipated through the cell bending, buckling, or fracture. [50]

Furthermore, it was concluded that the density of the memory foam had no significant effect on the change in the impact force, but as expected, higher densities will cause a reduction of impact force. This conclusion was also confirmed by Sounik et al. [49], who investigated the properties of polyurethane energy-absorbing (EA) foams and concluded that for the same thickness of 13 mm, foams of different densities reached maximum deceleration practically at the same time. They concluded that foams of different densities had similar energy absorption properties. The best explanation for such behavior can be found in [50]. Avalle et al. stated that polyurethane foams have a large intermediate plateau phase and very low sensitivity to the strain-rate. For these reasons, an increase in the relative density does not have a significant influence on their mechanical properties [50].

Although the influence of density is low, it is evident that the best test results were achieved with the use of memory foam with a density of 100 kg/m^3^ and a thickness of 20 mm. The test results for such memory foams and different composite plates are shown in Figure 11. For the glass fiber and polyester resin composite test plate, the lowest impact force of 5143.19 ± 237.65 N was measured, which was about 20% to 22.5% lower compared to other two test plates. This combination of composite plate and polyurethane foam showed the best absorption properties.

The largest deviations in the values of the results when looking at the individual force measurements with different memory foam parameters were visible with the glass fiber composites (e.g., for 80 kg/m^3^/15 mm, mean ± SD was 7154.86 ± 422.46) (Table 2). The reason for such a large standard deviation could be due to the residual air between the woven fibers and the possible imperfections in the production process. The plates did not crack during impact testing, but if the global measurements together with the hybrid composites were analyzed, the carbon/glass composite had a SD of 164.3 (Table 12) and the *r*^2^ was almost close to 1 (0.9947), which meant that the chosen model followed the data perfectly.

Interesting results were measured for the composite plates in combination with the memory foam with a density of 60 kg/m^3^ and a thickness of 10 mm. Only in the case of the glass fiber plate was a force reduction noticed, while in the case of the hybrid plates, the impact force measured was higher than the force measured on the same plate without the memory foam.

Based on the measured masses of the composite plates, the results of the mechanical properties obtained from the literature [38,39,40] and the production costs, a hybrid composite made of a combination of carbon and glass fibers would be an optimal replacement for a metal bumper. Furthermore, from the obtained results and price aspect, it is obvious that the second hybrid composite plate (carbon/aramid fiber and epoxy resin) showed the worst results and is not recommended for further research in the case of composite bumpers.

#### Optimization

The experiment revealed the factors that most influenced the force reduction and energy absorption. Software package *Design Expert* also includes an optimization module, in which optimization can be performed based on the desirability functions.

The optimization process searches for a combination of factor values that simultaneously satisfy the criteria (wishes and priorities) placed on each of the responses and factors [46].

In this research, optimization was performed for a hybrid composite (carbon/glass/epoxy) with memory foam. The optimization criteria (for optimal solution) were minimum density and minimum thickness of memory foam and maximum reduction of impact force.

Optimization constraints and criteria are given in Table 14.

For the given optimization conditions, only one solution was found, which is given in Table 14. Desirability *d* = 0.6216 resulting from optimization did not completely satisfy the set criteria. The minimum density condition is completely satisfied, while the thickness and force conditions *F* are not, so it could be expected that the desirability function would be slightly worse.

**Figure 12:**
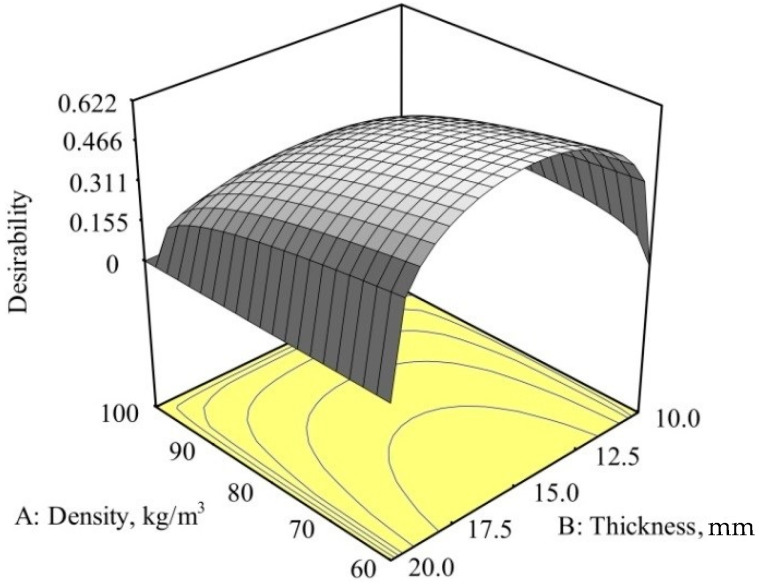
Desirability of force for the carbon/glass/epoxy hybrid composite.

The impact force dependence on the density and thickness of the memory foam within the optimization constraints is shown in Figure 12.

## 5. Conclusions

In recent years, the use of polymer composites in the automotive industry has been steadily increasing. Polymer composites have good mechanical properties and low mass, which are beneficial in the overall vehicle mass reduction and improvement in fuel efficiency.

Energy absorption properties of fiber-reinforced polymer composites combined with the memory foam were investigated in this paper. The impact force test on different types of composite test plates without the use of memory foam showed that the best results were achieved with the hybrid carbon/glass fiber composite and epoxy resin. With the application of additional absorbent material in the form of memory foam, significantly better absorption of the impact force was achieved with the reduction of 30% to 46%.

The lowest impact force, thus the best absorption of impact energy, was achieved with the use of memory foam with a density of 100 kg/m^3^ and a thickness of 20 mm in combination with the glass fiber composite plate. The analysis of the test results concluded that the thickness of the memory foam was the main factor affecting the reduction of the impact force.

The demands in the transport industry are increasing day by day. Customer wishes include high safety, better performance, and low costs. Furthermore, lower emissions and fuel consumption are required, and they are closely linked to the vehicle mass. All of these require the development of new materials, but as described in our work, perhaps it could be done not with completely new materials, but rather with new combinations of different types of existing materials.

Due to this, future studies should include different combinations of glass fiber and carbon fiber composites with different numbers of layers, different arrangements, and orientations of the fibers. In addition, energy absorption tests of composite plates and memory foams should be conducted with impactors of different shapes and masses, which should influence the energy absorption.

The experimental set-up and method will also be used to test energy absorption in other industries. The combination of composites and memory foam can be applied in the military industry for the production of protective vests and helmets. The most important point is that with composites, memory foam absorbs the energy and distributes projectile impact energy to the entire surface of the vest.

## Figures and Tables

**Table 1:** Arrangement of fibers.

	1. Plate (Glass Fiber)		2. Plate (Carbon/Aramid)		3. Plate (Glass/Carbon)	
Layer Number	Fiber Type	Rotation of Fiber Layer	Fiber Type	Rotation Fiber Layer	Fiber type	Rotation of Fiber Layer
1.	glass	0°	carbon	0°	carbon	0°
2.	glass	0°	carbon	0°	carbon	0°
3.	glass	0°	carbon	45°	glass	0°
4.	glass	0°	carbon	45°	glass	0°
5.	glass	0°	aramid	0°	carbon	0°
6.	glass	0°	aramid	0°	carbon	0°
7.			aramid	0°	glass	0°
8.			aramid	0°	glass	0°
9.			carbon	45°		
10.			carbon	45°		
11.			carbon	0°		
12.			carbon	0°		

**Table 2:** Results of the measured force for glass fiber/polyester resin and memory foam.

Memory Foam Density/Thickness kg/m^3^/mm	Glass Fiber/Polyester Resin + Memory Foam							
	80/15	60/20	80/15	60/10	80/15	100/20	100/10	-
No.	1	2	3	4	5	6	7	8
Force *F*, N	6874.85	5503.41	7286.87	8581.79	7494.84	4767.66	8338.5	9908.1
	7204.46	5473.98	6863.08	8281.60	7578.23	5279.74	8201.16	9638.33
	7332.98	5603.47	6869.94	8583.75	6788.52	5061.96	7345.73	8946.72
	7524.27	5150.25	6710.04	8495.46	7269.21	5368.03	7910.78	9653.04
	7425.19	5032.53	7539.97	8911.40	6633.52	5238.54	8175.65	9962.06
Mean $\bar{x}$, N	7272.35	5352.73	7053.98	8570.80	7152.86	5143.19	7994.37	9621.65
St. dev. *S*	251.55	246.88	346.06	226.72	422.46	237.65	394.26	404.58

**Table 3:** Results of the variance analysis for the glass fiber/polyester.

	Sum of Squares	Degrees of Freedom DF	Mean Square	*F* Value	Risk of Rejection of *H*_o_ (Prob > *F*)
Model	9.397 × 10^6^	3	3.132 × 10^6^	438.77	0.0351 significant
A	1.544 × 10^5^	1	1.544 × 10^5^	21.63	0.1348
B	9.209 × 10^6^	1	9.209 × 10^6^	1289.96	0.0177
AB	3.365 × 10^4^	1	3.365 × 10^4^	4.71	0.2748
Curvature	2.668 × 10^5^	1	2.669 × 10^5^	37.37	0.1032 not significant
Pure error	7.138 × 10^3^	1	7.138 × 10^3^		
Cor Total	9.671 × 10^6^	5			

**Table 4:** Overview of the statistical data about the model for glass fiber/polyester.

	Force, *F* (N)
Standard deviation	84.49
Mean	6914.38
Coefficient of determination *r*^2^ (R-squared)	0.9992

**Table 5:** Results of the standard error and confidence intervals.

Factor	Coefficient Estimate	DF	Standard Error	95% CI Low	95% CI High	VIF
Intercept	6765.27	1	42.25	6228.48	7302.06	
A-density	−196.49	1	42.25	−733.28	340.30	1
B-thickness	−1517.31	1	42.25	−2054.10	−980.52	1
AB	91.72	1	42.25	−445.07	628.51	1
Centre Point	447.33	1	73.17	−482.41	1377.08	1

**Table 6:** Results of measured force for the carbon and aramid fibers with epoxy resin and memory foam.

Memory Foam Density/Thickness kg/m^3^/mm	Carbon/Aramid Fibers/Epoxy Resin + Memory Foam							
	80/15	60/20	80/15	60/10	80/15	100/20	100/10	-
No.	1	2	3	4	5	6	7	8
Force *F*, N	8870.20	6553.08	8717.17	9770.76	8110.91	6715.93	9422.51	9378.36
	8699.51	6780.67	8711.28	9829.62	8152.11	6521.69	9429.37	9770.76
	8846.66	6749.28	6825.80	9922.82	8191.35	6665.90	9323.42	9594.18
	8558.24	6574.66	8593.56	9776.65	8252.17	6695.33	9158.62	9633.42
	8381.66	7043.58	8358.12	10094.49	8063.82	6603.11	9405.83	9339.12
Mean $\bar{x}$, N	8671.26	6740.25	8241.18	9878.87	8154.07	6640.39	9347.95	9543.17
St. dev., *S*	204.80	197.60	167.89	135.08	60.12	78.83	114.0	181.21

**Table 7:** Variance analysis results for the carbon/aramid/epoxy hybrid composite.

	Sum of Squares	Degrees of Freedom DF	Mean Square	*F* Value	Risk of Rejection of *H*_o_ (Prob > *F*)
Model	8.690 × 10^6^	3	2.897 × 10^6^	40.85	0.0240 significant
A	9.947 × 10^4^	1	9.947 × 10^4^	1.40	0.3580
B	8.544 × 10^6^	1	8.544 × 10^6^	120.48	0.0082
AB	4.645 × 10^4^	1	4.645 × 10^4^	0.66	0.5033
Curvature	1.857 × 10^5^	1	1.857 × 10^5^	2.62	0.2470 not significant
Pure error	1.418 × 10^5^	2	7.092 × 10^4^		
Cor Total	9.018 × 10^6^	5			

**Table 8:** Overview of the statistical data about the model for the carbon/aramid/epoxy hybrid composite.

	Force, *F* (N)
Standard deviation	266.31
Mean	8292.91
Coefficient of determination *r*^2^ (R-squared)	0.9839

**Table 9:** Results of the standard error and confidence intervals.

Factor	Coefficient Estimate	DF	Standard Error	95% CI Low	95% CI High	VIF
Intercept	8151.87	1	133.15	7578.96	8724.77	
A-density	−157.70	1	133.15	−730.60	415.21	1
B-thickness	−1461.55	1	133.15	−2034.45	−888.64	1
AB	107.77	1	133.15	−465.14	680.67	1
Centre Point	329.11	1	203.39	−546.02	1204.24	1

**Table 10:** Results of the measured force for the carbon and glass fiber with epoxy resin and memory foam.

Memory Foam Density/Thickness kg/m^3^/mm	Carbon/Glass Fiber/Epoxy Resin + Memory Foam							
	80/15	60/20	80/15	60/10	80/15	100/20	100/10	-
No.	1	2	3	4	5	6	7	8
Force *F*, N	8622.99	6132.23	8583.75	9711.9	8517.04	6543.27	9579.47	9405.83
	8128.57	6662.95	8730.9	9682.47	8546.47	6333.34	9613.8	9309.69
	8393.44	6626.66	8913.37	9594.18	8499.38	6445.17	9488.23	9262.60
	8564.13	6562.89	8721.09	9732.50	8505.27	6180.3	9588.29	9417.6
	8456.22	6538.37	8784.86	9500.0	8458.18	6380.42	9641.29	9199.82
Mean $\bar{x}$, N	8433.07	6504.62	8746.79	9644.21	8505.27	6376.5	9582.21	9319.11
St. dev., *S*	192.41	214.0	119.05	96.36	31.97	135.0	57.80	93.18

**Table 11:** Results of the variance analysis for carbon/glass/epoxy hybrid composite.

	Sum of Squares	Degrees of Freedom DF	Mean Square	*F* Value	Risk of Rejection of *H*_o_ (Prob > *F*)
Model	1.001 × 10^7^	3	3.359 × 10^6^	124.42	0.0080 significant
A	9.036 × 10^3^	1	9.036 × 10^3^	0.33	0.6213
B	1.007 × 10^7^	1	1.007 × 10^7^	372.88	0.0027
AB	1.092 × 10^3^	1	1.092 × 10^3^	0.04	0.8591
Curvature	4.904 × 10^5^	1	4.904 × 10^5^	18.17	0.0509 not significant
Pure error	5.399 × 10^4^	2	2.699 × 10^4^		
Cor Total	1.062 × 10^7^	5			

**Table 12:** Overview of the statistical data about the model for the carbon/glass/epoxy hybrid composite.

	Force, *F* (N)
Standard deviation	164.30
Mean	8256.10
Coefficient of determination *r*^2^ (R-squared)	0.9947

**Table 13:** Results of the standard errors and confidence intervals.

Factor	Coefficient Estimate	DF	Standard Error	95% CI Low	95% CI High	VIF
Intercept	8026.89	1	82.15	7673.42	8380.35	
A-density	−47.53	1	82.15	−400.99	305.93	1
B-thickness	−1586.33	1	82.15	−1939.79	−1232.86	1
AB	−16.53	1	82.15	−369.99	336.93	1
Centre Point	534.82	1	125.49	−5.10	1074.75	1

**Table 14:** Constraints and optimization solution.

Name	Goal	Lower Limit	Upper Limit	Solution
Density, kg/m^3^	minimize	60	100	60
Thickness, mm	minimize	10	20	15
Force, N	minimize	6376.5	9644.21	8073.57
